# Change of mortality of patients with acute ischemic stroke before and after 2015

**DOI:** 10.3389/fneur.2022.947992

**Published:** 2022-08-24

**Authors:** Sang-Won Park, Ji Young Lee, Nam Hun Heo, James Jisu Han, Eun Chae Lee, Dong-Yong Hong, Dong-Hun Lee, Byung Cheol Lee, Young Wha Lim, Gui Ok Kim, Man Ryul Lee, Jae Sang Oh

**Affiliations:** ^1^Department of Neurosurgery, College of Medicine, Soonchunhyang University, Cheonan Hospital, Cheonan, South Korea; ^2^Department of Molecular Biophysics and Biochemistry, Yale University, New Haven, CT, United States; ^3^Health Insurance Review and Assessment Service (HIRA), Chuncheon, South Korea; ^4^Soonchunhyang Institute of Medi-Bio Science (SIMS), Soonchunhyang University, Cheonan, South Korea

**Keywords:** stroke, acute ischemic stroke, mechanical thrombectomy (MT), intravenous thrombolysis (IVT), trend research, stroke care

## Abstract

**Introduction:**

Advances in the diagnosis and management of acute ischemic stroke (AIS) and the increased use of mechanical thrombectomy (MT) have improved the quality of care and prognosis of patients with AIS since 2015. We investigated the changing trends in mortality of patients with AIS in Korea before and after 2015.

**Materials and methods:**

A retrospective cohort study was conducted using combined anonymized data from the Acute Stroke Assessment Registry of Korea and the Health Insurance Review & Assessment Service database. Patients with ischemic stroke with precise onset time and initial National Institute of Health Stroke Scale records were included.

**Results:**

Patients receiving MT treatment increased from 256 (2.7%) pre-2015 to 1,037 (3.9%) post-2015 (*p* < 0.001). Overall mortality significantly decreased from pre-2015 to post-2015. In pre-2015, intravenous thrombolysis (IVT) administered within 2 h significantly reduced 3-month mortality when compared with non-IVT. While, in post-2015, IVT administered within 2 h significantly reduced the 3-month, 1-year, 2-year, and 4-year mortality (*p* < 0.05). MT only reduced 1-year mortality pre-2015; however, MT significantly reduced the 3-month, 1-year, and 2-year mortality post-2015 (*p* < 0.05). Post-stroke antiplatelet and anticoagulant drugs significantly reduced the 3-month, 1-year, 2-year, and 4-year mortality post-2015.

**Discussion:**

Since 2015, faster IVT has significantly reduced the short- and long-term mortality in patients with AIS; MT reduced the 3-month, 1-year, and 2-year mortality. Post-stroke antithrombotic medication has significantly lowered the 2- and 4-year mortality since 2015.

**Conclusions:**

Changing trends in AIS management since 2015 have improved the prognosis of patients with AIS.

## Introduction

Stroke is the leading cause of death, which is the second most common cause of death and disability worldwide (1, 2). Ischemic stroke accounted for 62.4% of all strokes, and its age-standardized incidence showed less reduction than that of intracerebral and subarachnoid hemorrhage from 1990 to 2019 (3). Since the American Heart Association/American Stroke Association updated its guidelines in 2015, mechanical thrombectomy (MT) has become the primary strategy along with intravenous thrombolysis (IVT) (4). Korea established the usefulness of MT and has changed the health insurance coverage of MT since 2015. The indication of MT largely depended on the operator's individual opinion prior to 2015. After MT was approved for insurance in Korea in 2015, the indications for MT became more accurate within 8 h, and the Health Insurance Review & Assessment Service (HIRA) recommended MT for only selected patients with AIS, according to image findings.

Some reports stated that IVT lowers long-term mortality (5–8). However, our previous study using this multicenter large observation data has found that IVT had decreased the 3-month mortality of patients with AIS before 2015 (9), but long-term (1- and 5-year) mortality could not be lowered. According to change of paradigm about AIS management in 2015, we need to study about the changing trend of the prognosis of patients with AIS before and after 2015. So, we investigated the short- and long-term mortality of patients with AIS from 2013 to 2018.

## Materials and methods

Korea developed the nationwide Acute Stroke Assessment Registry (ASAR) to control the quality of acute stroke care in 2011, and this registry was performed for 3 months each year. These 3 months were selected by HIRA from 2007 to 2014 and 6 months from 2016 onwards to assess healthcare quality. In Korea, an acute stroke registry database is being established, examined, and graded by HIRA and is used for analysis. These ASAR data are collected once every 2-year (201 hospitals that met certain conditions in 2013, 189 hospitals in 2014, 246 hospitals in 2016, and 248 hospitals in 2018). Also, all patients' data between 2011 and 2022 were collected by HIRA.

Under the government research project, we linked the ASAR and HIRA databases to collect patients' clinical and management data and evaluate patient outcomes. Furthermore, this is a reliable data source to assess the quality of acute stroke care and monitor long-term follow-up outcomes (10).

### Inclusion and exclusion criteria

**Figure 1:**
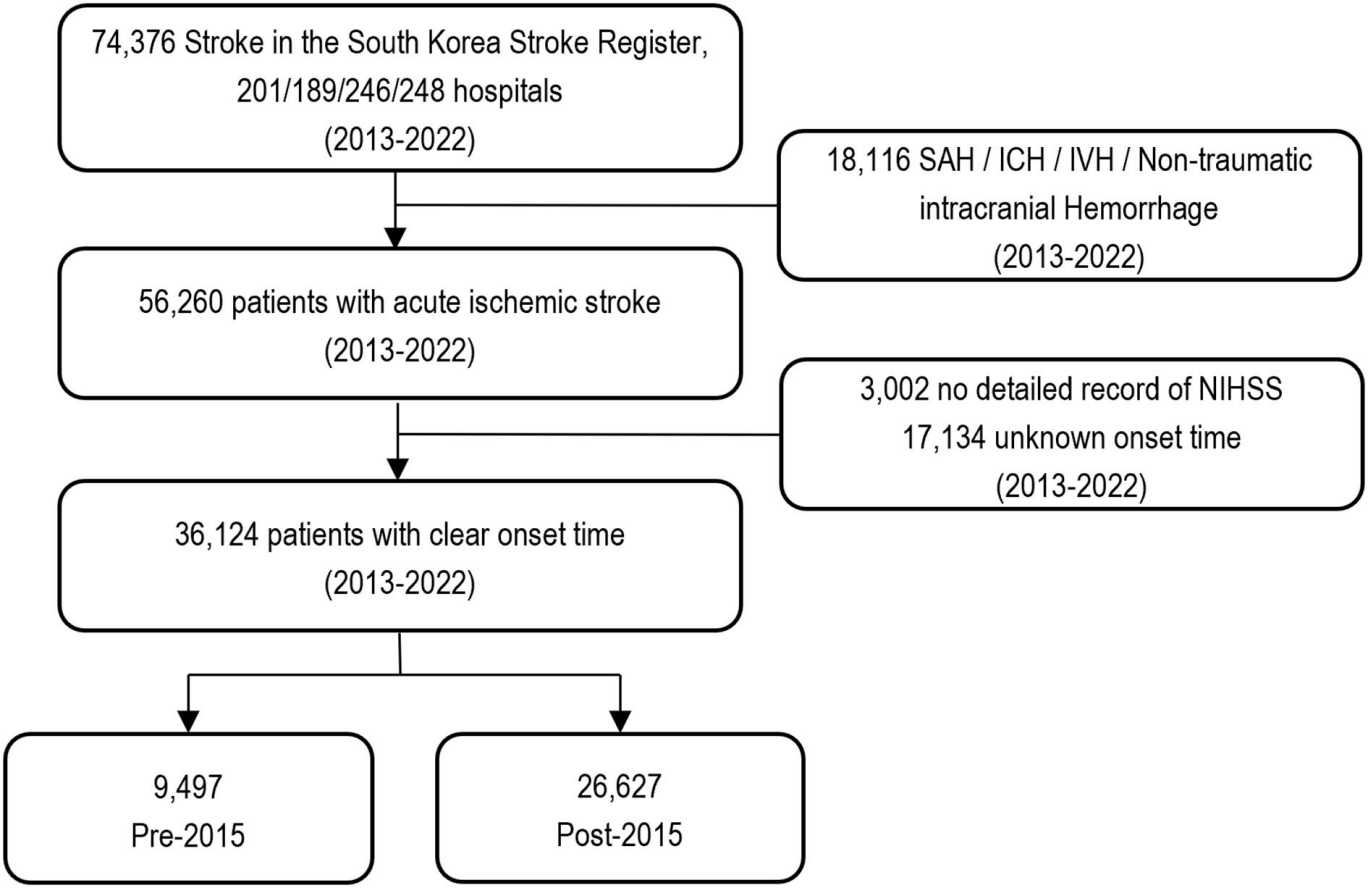
A flow chart of study population. NIHSS, National Institutes of Health Stroke Scale; SAH, subarachnoid hemorrhage; ICH, intracerebral hemorrhage; IVH, intraventricular hemorrhage.

All patients admitted to stroke centers with a main diagnosis of first-ever AIS examined within 24 h of admission were selected from the ASAR from March to May 2013, June to August 2014, July to December 2016, and July to December 2018. Of these, 3,002 patients with no National Institute of Health Stroke Scale (NIHSS) scores and 17,134 patients with no precise onset time were excluded from this study to observe changes in treatment effectiveness (Figure 1). Overall, 36,124 patients were included, and data were collected on the duration of hospital stay, thrombectomy, medication, ICD-10 codes, and death from HIRA (Supplementary Table 1). All data were anonymized using encrypted personal identification numbers in partnership with HIRA under the Joint Project on Quality Assessment Research in Korea. This study was approved by the Research Ethics Committee of Soonchunhyang University Hospital (IRB No. SCHCH 2021-01-007). The need for informed consent was waived because of the study's retrospective nature.

### Clinical and management data

The following demographic and clinical data were collected from the stroke registry: age, sex, stroke type, economic status (insurance type), time (the symptom onset, onset-to-door, onset-to-image, onset-to-needle), arrival mode, NIHSS, medical history, smoking history, type of medical facility, stroke unit, thrombolysis, and functional outcome. Other information collected from HIRA included thrombectomy, presence of comorbidities (atrial fibrillation, cardiovascular risk factors, and other diseases), medication information before admission, and death.

Age at the onset of ischemic stroke was calculated as the difference between the date of the symptom onset and the recorded date of birth. Health insurance coverage was stratified by economic status: standard South Korean health insurance and low-income medical aid. Onset-to-door time was calculated as the difference between symptom onset time and recorded time of hospital arrival; door-to-image time was calculated as the difference between hospital arrival time and brain imaging time; onset-to-needle time was calculated as the difference between the symptom onset and IVT-treated time. Arrival mode was divided according to the use or non-use of emergency medical services (EMS) before hospital arrival. The initial NIHSS score was stratified into five groups: 1–4, 5–7, 8–13, 14–21, and 22–42 (11). The Charlson Comorbidity Index (CCI) was calculated using ICD-10 codes and stratified into four levels: 0, 1, 2, and ≥ 3.

Medical facilities were divided into two groups (tertiary and general hospitals), and hospitals were divided into hospitals with and without stroke units. HIRA uses absolute evaluation (treatment function, educational function, manpower/facility/equipment, patient composition ratio, medical service level) and relative evaluation of general and tertiary hospitals to calculate and determine the appropriate number of beds for each treatment area. Stroke units are selected as national accreditation centers by accrediting hospitals with adequate hospital personnel, access to multidisciplinary teams, systematic and structured diagnosis and treatment, and monitoring of clinical status and vital signs through accreditation bodies. The IVT dosage depends on the patient's weight; the approved dosage ranges from.9 mg/kg to 90 mg (12). MT was performed to recanalize the large-artery occlusion after IVT. Post-stroke antithrombotic medication use was defined as taking antithrombotics for at least 1 week after stroke and was divided into antiplatelet and anticoagulant medication. Antithrombotic drugs were prescribed as follows: antiplatelet = aspirin, cilostazol, clopidogrel, ticlopidine; anticoagulant = direct-acting oral anticoagulant (DOAC), warfarin. The development of intracranial hemorrhage (ICH) was defined as its occurrence (ICD-10: I61) in patients with AIS on admission (within 30 days) after MT.

### Study grouping

The patients were divided into two groups: pre-2015— patients with AIS before 2015 (*n* = 9,497, 26.3%), and post-2015— patients with AIS after 2015 (*n* = 26,627, 73.7%).

In addition to the overall outcome, we assessed the impact of IVT and MT. The patients with AIS treated with IVT were stratified into three groups: IVT received within 2 h after the symptom onset, 2 h and 4.5 h after the symptom onset, and non-IVT, while patients with AIS were stratified into two groups according to whether they received MT (MT and non-MT).

### Outcome

Using de-identified patient codes, we combined acute stroke registry data with patients' survival data on HIRA to track patients during our follow-up period. All patients included in this study were followed for 4 years after the stroke onset. The functional outcome at discharge was defined as good (75–99 points on the Korean Version of Modified Barthel Index, Modified Barthel Index, or Barthel Index); 90 or higher on the Functional Independence Measure; two or lower on the modified Rankin Scale); or a Glasgow Outcome Scale score of 5 or poor (13–16).

### Statistical analysis

Pre-and post-2015 patient data were compared using the independent *t*-test for continuous variables [expressed as mean ± standard deviation (SD)] and the chi-squared tests for categorical variables [expressed as percentages (counts)]. NIHSS scores were expressed as mean (SD) in each group. Primary outcomes analyzed were survival at 3 months, 1 year, 2 years, and 4 years after the symptom onset. We reported Kaplan–Meier (KM) survival estimates, and the difference between survival curves was tested using the log-rank test. All patients were tracked for 4 years, and those who survived until the end of the tracking period (April 15, 2021) were defined as censored data.

Multivariable Cox proportional hazard models were fitted to pre- and post-2015 data to estimate hazard ratios (HR) and 95% confidence intervals (CI) for covariates to match the patients' conditions equally. In addition, Cox analysis was conducted to determine the treatment effect when other variables were controlled. The models included onset-to-needle time, implementation of MT, age, sex, health insurance type, arrival mode, NIHSS score, door-to-image time, medical history, CCI: medical facility type, and the use of post-stroke antithrombotic medication (antiplatelet or anticoagulant medication) as covariates. A proportional hazards assumption was used to validate the application of Cox proportional hazard models. Data analysis was performed using SAS version 9.3 (SAS Institute, Cary, NC) and R version 3.5.1 (The R Foundation for Statistical Computing, Vienna, Austria). A two-sided test with *p* < 0.05 was considered statistically significant.

## Results

### Comparison between pre- and post-2015 groups

Table 1 shows the comparison between pre-2015 and post-2015. The mean patient ages were 67.9 ± 12.5 years and 68.5 ± 13. years in pre- and post-2015, respectively (*p* < 0.0001). Males accounted for a significantly higher proportion than females in both groups [males: 59.1% [5,612], females: 40.9% (3,885) in pre-2015; males: 59.5% (15,835), females: 40.5% (10,792) in post-2015; *p* < 0.001]. The proportions of sexes in pre- and post-2015 were not significantly different (*p* = 0.52). The mean NIHSS score in pre-2015 was significantly higher than that in post-2015 (4.8 ± 5.6 vs. 4.6 ± 5.4, *p* = 0.004).

**Table 1:** Baseline characteristics of 36,124 patients with acute ischemic stroke between pre-2015 and post-2015 groups.

**Variables**	**Total**	**Pre-2015**	**Post-2015**	***p*-value**
Total	36,124 (100%)	9,497 (26.3%)	26,627 (73.7%)	
Age, mean (SD), y	68.4 ± 12.9	67.9 ± 12.5	68.5 ± 13.0	<0.001
18–45 years (n, %)	7,469 (20.7%)	1,857 (19.6%)	5,612 (21.0%)	0.003
46–59	7,514 (20.8%)	2,038 (21.5%)	5,476 (20.6%)	
60–69	6,598 (18.3%)	1,804 (19.0%)	4,794 (18.0%)	
≥70	14,543 (40.3%)	3,798 (40.0%)	10,745 (40.4%)	
Male, sex	21,447 (59.4%)	5,612 (59.1%)	15,835 (59.5%)	0.520
Female, sex	14,677 (40.6%)	3,885 (40.9%)	10,792 (40.5%)	
Health insurance type				
Health insurance	31,719 (93.2%)	7,756 (93.6%)	23,968 (93.0%)	0.106
Medical aid	2,326 (6.8%)	534 (6.4%)	1,792 (7.0%)	
NIHSS, mean (SD)	4.7 ± 5.5	4.8 ± 5.6	4.6 ± 5.4	0.002
NIHSS, IQR	3 (1–6)	3 (1–6)	3 (1–6)	<0.001
1–4	23,575 (65.3%)	6,272 (66.0%)	17,303 (65.0%)	<0.001
5–7	5,726 (15.9%)	1,398 (14.7%)	4,328 (16.3%)	
8–13	3,744 (10.4%)	960 (10.1%)	2,784 (10.5%)	
14–21	2,368 (6.6%)	644 (6.8%)	1,724 (6.5%)	
22–42	711 (2.0%)	223 (2.4%)	488 (1.8%)	
Medical history				
Smoker				
Current smoker (*n*, %)	5,534 (25.3%)	2,492 (26.4%)	3,042 (24.4%)	<0.001
Ex-smoker	2,903 (13.2%)	1,393 (14.7%)	1,510 (12.1%)	
Non-smoker	13,478 (61.5%)	5,571 (58.9%)	7,907 (63.5%)	
Atrial fibrillation/flutter	5,686 (16.1%)	1,474 (16.0%)	4,212 (16.2%)	0.623
CCI score				
0	7,469 (20.7%)	1,857 (19.5%)	5,612 (21.1%)	0.002
1	7,514 (20.8%)	2,038 (21.5%)	5,476 (20.6%)	
2	6,598 (18.2%)	1,804 (19.0%)	4,794 (18.0%)	
≥3	14,543 (40.3%)	3,798 (40.0%)	10,745 (40.3%)	
Arrival mode (*n*, %)				
EMS	17,647 (48.9%)	4,468 (47.0%)	13,179 (49.5%)	<0.001
No EMS	18,477 (51.1%)	5,029 (53.0%)	13,448 (50.5%)	
Onset-to-door time (*n*, %)				
≤ 2 h	10,200 (28.2%)	2,732 (28.8%)	7,468 (28.0%)	0.181
>2 h	25,924 (71.8%)	6,765 (71.2%)	19,159 (72.0%)	
Door-to-image time (n, %)				
≤ 1 h	27,586 (88.2%)	6,812 (82.9%)	20,774 (90.1%)	<0.001
>1 h	3,688 (11.8%)	1,407 (17.1%)	2,281 (9.9%)	
Hospital level				
Medical facility type (*n*, %)				
Tertiary general hospital	17,906 (49.6%)	4,990 (52.5%)	12,916 (48.5%)	<0.001
General hospital	18,218 (50.4%)	4,507 (47.5%)	13,711 (51.5%)	
Stroke unit (*n*, %)				
Yes	22,530 (62.4%)	5,851 (61.6%)	16,679 (62.6%)	0.075
No	13,594 (37.6%)	3,646 (38.4%)	9,948 (37.4%)	
IVT				
Onset-to-needle time (min)	125.7 ± 55.3	121.1 ± 50.9	127.1 ± 56.8	<0.001
IVT ≤ 120 min	2,269 (6.3%)	622 (6.5%)	1,647 (6.2%)	0.017
120 min < IVT ≤ 270 min	1,867 (5.2%)	537 (5.7%)	1,330 (5.0%)	
Non-IVT	31,988 (88.6%)	8,338 (87.8%)	23,650 (88.8%)	
Mechanical thrombectomy (*n*, %)	1,293 (3.6%)	256 (2.7%)	1,037 (3.9%)	<0.001
Post antithrombotic medication (*n*, %)				
No medication	2,573 (7.1%)	1,339 (14.1%)	1,234 (4.6%)	<0.001
Antiplatelet medication	27,331 (75.7%)	6,557 (69.0%)	20,774 (78.0%)	
Anticoagulant medication	2,021 (3.3%)	469 (4.9%)	1,552 (5.8%)	
Antiplatelet & Anticoagulant	4,199 (11.6%)	1,132 (11.9%)	3,067 (11.5%)	
Functional outcome at discharge			
Good outcome	23,645 (66.0%)	6,376 (67.3%)	17,269 (65.5%)	0.002
Poor outcome	12,184 (34.0%)	3,099 (32.7%)	9,085 (34.5%)	
mRS				
0	4,868 (17.2%)	1,279 (17.6%)	3,589 (17.1%)	<0.001
1	9,727 (34.5%)	2,559 (35.3%)	7,168 (34.2%)	
2	5,313 (18.8%)	1,316 (18.1%)	3,997 (19.1%)	
3	3,581 (12.7%)	825 (11.4%)	2,756 (13.1%)	
4	2,891 (10.2%)	708 (9.8%)	2,183 (10.4%)	
5	1,556 (5.5%)	425 (5.9%)	1,131 (5.4%)	
6	296 (1.1%)	140 (1.9%)	156 (0.7%)	
No. of death, n (%)				
3-month	1,911 (5.3%)	553 (5.8%)	1,358 (5.1%)	0.007
1-year	3,754 (10.4%)	1,077 (11.3%)	2,676 (10.1%)	<0.001
2-year	5,483 (15.2%)	1,534 (16.2%)	3,941 (14.8%)	0.002
4-year	7,627 (21.1%)	2,294 (24.2%)	5,333 (20.0%)	<0.001

*Values are presented as mean ± SD or n (%)*.

*NIHSS were presented as median and interquartile range (IQR)*.

*SD, standard deviation; NIHSS, National Institutes of Health Stroke Scale; IQR, Interquartile range; EMS, emergency medical services; CCI, Charlson comorbidity index; IVT, intravenous thrombolysis; mRS, modified Rankin Scale*.

*Good Outcome: K-MBI (75–99), MBI (75–99), BI (75–99), mRS (0–2), FIM (90–126), GOS (5)*.

*The patients who had no record of a functional outcome at discharge were excluded*.

The use of post-stroke antithrombotics in patients with AIS increased significantly post-2015 (*p* < 0.001). The proportion of patients who were not prescribed any antithrombotic medication decreased from 14.1% (1,339) in pre-2015 to 4.6% (1,234) in post-2015. In pre-2015, 69.% (6,557) of patients were prescribed antiplatelet medications compared to 78.% (20,774) in post-2015, while 4.9% (469) of patients were prescribed anticoagulant medications in pre-2015 compared to 5.8% (1,552) in post-2015; and 11.9% (1,132) of patients were prescribed both antiplatelet and anticoagulant medication in pre-2015 compared to 11.5% (3,067) in post-2015. Overall, 6,376 (67.3%) and 17,269 (65.5%) patients were discharged with a good outcome in pre- and post-2015 groups, respectively.

### Analysis of AIS patient treatments pre- and post-2015

Overall, 9,497 (26.3%) patients with AIS were enrolled in pre-2015. In pre-2015, 47.2% (4,468) of patients arrived at hospital within 4.5 h of the symptom onset. Of these, 12.2% (1,159) received IVT: 6.5% (622) within 2 h, and the remaining 5.7% (537) within 4.5 h; 2.7% (256) received MT. A total of 26,627 (73.7%) patients with AIS were enrolled in post-2015. In post-2015, 42.2% (11,241) of the patients arrived at hospital within 4.5 h of the symptom onset. Of these, 11.2% (2,977) received IVT: 6.2% (1,647) within 2 h, and 5.0% (1,330) within 4.5 h; 3.9% (1,037) received MT.

No significant difference was observed in the proportion of patients prescribed cilostazol [1,159 (12.2%) to 3,277 (12.3%), respectively] when comparing before and after 2015. In contrast, there were significant increases in the numbers of patients prescribed clopidogrel [5,066 (53.3%) to 18,301 (68.7%), respectively], aspirin [6,716 (70.7%) to 21,700 (81.5%), respectively], and DOAC [78 (0.8%) to 3,616 (13.8%), respectively]. There were significant decreases in the numbers of patients prescribed ticlopidine [388 (4.1%) to 783 (2.9%), respectively] and warfarin [1,568 (16.5%) to 1,143 (4.3%), respectively] (Supplementary Table 2).

### Mortality pre- and post-2015

A total of 7,627 (21.1%) patients died during the 4-year follow-up period. The 3-month, 1-year, 2-year, and 4-year mortality rates were 5.3% (1,911), 10.4% (3,754), 15.2% (5,483), and 21.1% (7,627), respectively. Overall mortality in post-2015 decreased significantly compared with pre-2015; moreover, mortality in post-2015 decreased significantly from 5.8% (553) to 5.1% (1,358) at 3 months (*p* = 0.007), from 11.3% (1,077) to 10.1% (2,677) at 1 year (*p* = 0.0004), from 16.2% (1,536) to 14.8% (3,947) at 2 years (*p* = 0.002), and from 24.4% (2,294) to 20.% (5,333) at 4 years (*p* < 0.001) when compared with pre-2015.

In mortality according to treatment, the patients treated with IVT post-2015 had significantly lower 3-month (10.5 vs. 6.0%), 1-year (15.6 vs. 9.8%), 2-year (20.2 vs. 13.6%), and 4-year (28.1 vs. 18.%) mortality than pre-2015. Furthermore, those treated with MT post-2015 had significantly lower 3-month (19.5 vs.16.4%), 1-year (26.6 vs. 21.5%), 2-year (31.6 vs. 26.6%), and 4-year (39.8 vs. 31.4%) mortality than pre-2015.

**Figure 2:**
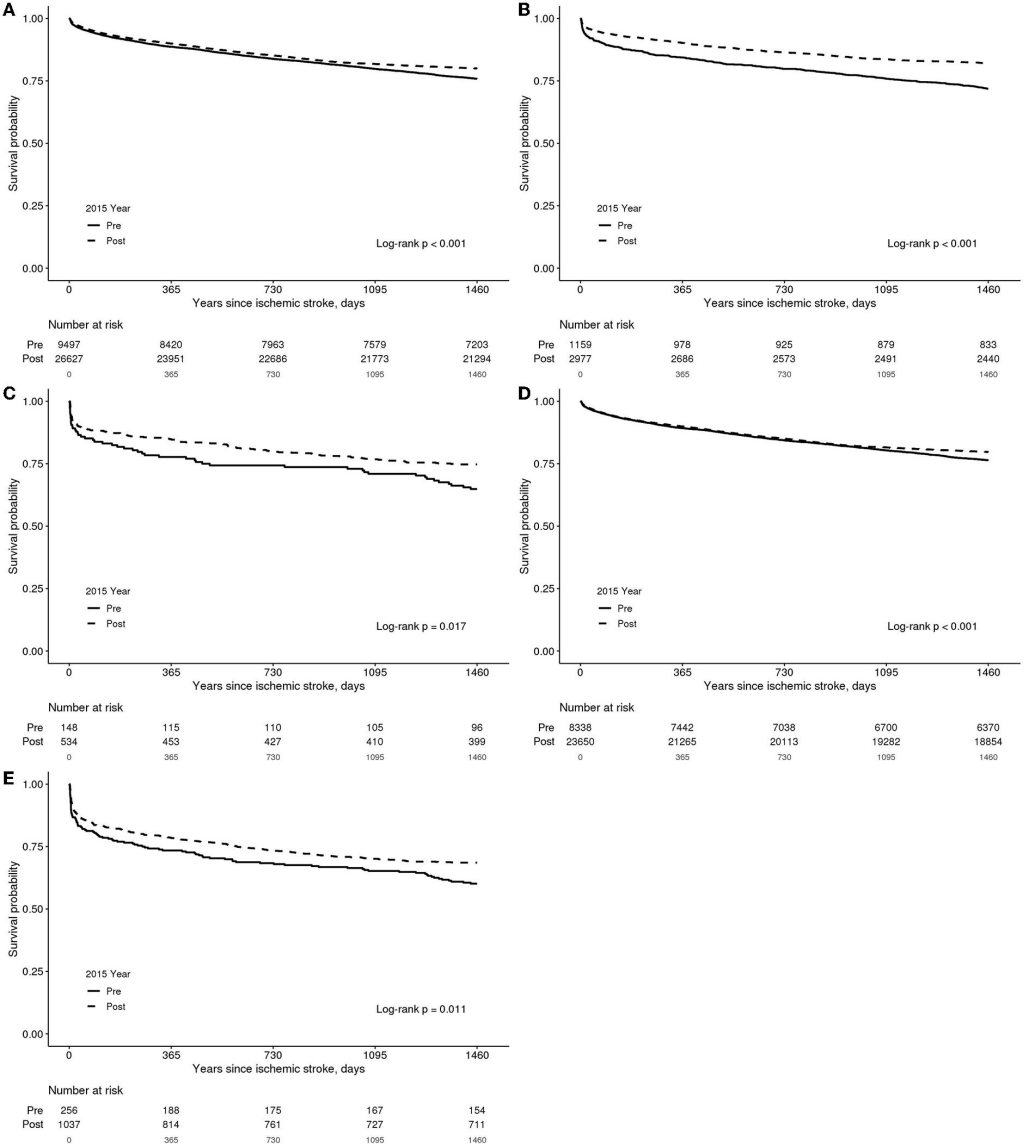
A Kaplan–Meier estimate in patients with acute ischemic stroke according to observation groups. **(A)** Total patients. **(B)** Patients who underwent intravenous thrombolysis. **(C)** Patients who underwent both intravenous thrombolysis and mechanical thrombectomy. **(D)** Patients who did not undergo intravenous thrombolysis. **(E)** Patients who underwent mechanical thrombectomy.

The KM survival analysis showed lower mortality post-2015 than pre-2015 (Figure 2A, the log-rank test, *p* < 0.001). The KM estimate for IVT showed significantly lower mortality in post-2015 than in pre-2015 (Figure 2B, the log-rank test, *p* < 0.001); the patients treated with both IVT and MT had significantly lower mortality in post-2015 than in pre-2015 (Figure 2C, the log-rank test, *p* = 0.017). The patients not receiving IVT had significantly lower mortality post-2015 (Figure 2D, the log-rank test, *p* < 0.001). Furthermore, those treated with MT had significantly lower mortality post-2015 (Figure 2E, the log-rank test, *p* < 0.001).

In the KM survival analysis of pre-2015 patients, and according to MT or non-MT in each NIHSS score, MT did not show a significant difference in mortality with NIHSS 1–4, 5–7, 8–13, or 22–42. In addition, there was no significant difference in mortality in the mild patient group of NIHSS 1-4 and 5–7 in post-2015; however, MT showed significantly lower mortality in the severe patient group of NIHSS 8–13, 14–21, and 22–42 (Supplementary Figures 1, 2, the log-rank test, *p* < 0.05).

### Cox analysis of mortality risk factors for AIS

Table 2 compares Cox regression on survival between pre- and post-2015.

**Table 2:** Comparison of multivariable cox regression on survival between pre-2015 and post-2015.

	**3-month**		**1-year**		**2-year**		**4-year**	
	**HR (95% CI)**		**HR (95% CI)**		**HR (95% CI)**		**HR (95% CI)**	
	**Pre-2015**	**Post-2015**	**Pre-2015**	**Post-2015**	**Pre-2015**	**Post-2015**	**Pre-2015**	**Post-2015**
Onset to treatment time								
IVT ≤ 120 min	0.62 (0.52–0.74)^‡^	0.53 (0.44–0.64)^‡^	0.82 (0.69–0.98)*	0.66 (0.55–0.79)^‡^	0.80 (0.67–0.95)*	0.77 (0.64–0.93)^†^	0.63 (0.53–0.75)^‡^	0.77 (0.64–0.93)^†^
120 min < IVT ≤ 270 min	0.72 (0.60–0.87)^‡^	0.70 (0.58–0.86)^‡^	0.83 (0.68–1.00)*	0.80 (0.66–0.98)*	0.82 (0.68–0.99)*	0.77 (0.63–0.94)*	0.93 (0.75–1.12)	0.63 (0.51–0.77)^‡^
Non–IVT	1.0	1.0	1.0	1.0	1.0	1.0	1.0	1.0
Mechanical Thrombectomy								
Yes	0.80 (0.63–1.00)	0.69 (0.56–0.84)^‡^	0.88 (0.70–1.11)	0.75 (0.61–0.92)^†^	0.95 (0.75–1.19)	0.80 (0.65–0.99)*	0.91 (0.72–1.14)	1.10 (0.89–1.36)
No	1.0	1.0	1.0	1.0	1.0	1.0	1.0	1.0
Age (years)								
18–45	1.0	1.0	1.0	1.0	1.0	1.0	1.0	1.0
46–59	2.49 (1.38–4.49)^†^	2.61 (1.32–5.15)^†^	2.52 (1.40–4.55)^†^	2.70 (1.37–5.32)^†^	2.47 (1.37–4.46)^†^	2.57 (1.30–5.06)^†^	2.32 (1.29–4.19)^†^	1.85 (0.94–3.60)
60–69	5.50 (3.09–9.79)^‡^	5.41 (2.78–10.52)^‡^	5.58 (3.14–9.93)^‡^	5.29 (2.72–10.29)^‡^	5.16 (2.90–9.18)^‡^	4.86 (2.50–9.45)^‡^	4.34 (2.44–7.73)^‡^	2.98 (1.53–5.82)^†^
≥70	15.64 (8.84–27.65)^‡^	18.23 (9.44–35.18)^‡^	14.07 (7.96–24.86)^‡^	15.05 (7.79–29.06)^‡^	11.69 (6.61–20.68)^‡^	11.12 (5.76–21.49)^‡^	6.86 (3.87–12.16)^‡^	3.19 (1.65–6.17)^‡^
Male, sex	1.0	1.0	1.0	1.0	1.0	1.0	1.0	1.0
Female, sex	0.78 (0.71–0.86)^‡^	0.83 (0.76–0.92)^‡^	0.76 (0.69–0.84)^‡^	0.98 (0.89–1.07)	0.82 (0.74–0.90)^‡^	0.96 (0.87–1.05)	0.91 (0.83–1.00)	1.04 (0.94–1.14)
Health insurance type								
Health insurance	1.0	1.0	1.0	1.0	1.0	1.0	1.0	1.0
Medical aid	1.35 (1.18–1.55)^‡^	1.20 (1.04–1.38)*	1.36 (1.19–1.56)^‡^	1.08 (0.94–1.25)	1.33 (1.16–1.53)^‡^	1.01 (0.88–1.16)	1.14 (0.99–1.30)	0.97 (0.84–1.12)
Arrival mode								
EMS	1.0	1.0	1.0	1.0	1.0	1.0	1.0	1.0
No EMS	0.84 (0.77–0.92)^‡^	0.74 (0.67–0.81)^‡^	0.81 (0.74–0.89)^‡^	0.84 (0.77–0.93)^‡^	0.88 (0.81–0.97)^†^	0.86 (0.78–0.94)^†^	0.83 (0.77–0.91)^‡^	0.85 (0.77–0.93)^‡^
NIHSS								
1–4	1.0	1.0	1.0	1.0	1.0	1.0	1.0	1.0
5–7	1.46 (1.31–1.64)^‡^	1.50 (1.33–1.68)^‡^	1.31 (1.17–1.47)^‡^	1.36 (1.22–1.53)^‡^	1.26 (1.13–1.42)^‡^	1.35 (1.20–1.52)^‡^	1.20 (1.07–1.34)^†^	1.10 (0.98–1.23)
8–13	1.84 (1.61–2.09)^‡^	2.04 (1.80–2.31)^‡^	1.49 (1.31–1.70)^‡^	1.75 (1.55–1.99)^‡^	1.74 (1.53–1.98)^‡^	1.38 (1.21–1.56)^‡^	1.40 (1.23–1.59)^‡^	1.36 (1.21–1.54)^‡^
14–21	2.55 (2.18–2.99)^‡^	2.41 (2.07–2.81)^‡^	1.89 (1.62–2.21)^‡^	2.01 (1.73–2.33)^‡^	1.78 (1.52–2.08)^‡^	1.59 (1.36–1.85)^‡^	2.07 (1.78–2.42)^‡^	1.56 (1.35–1.81)^‡^
22–42	3.77 (2.97–4.79)^‡^	3.43 (2.73–4.31)^‡^	2.68 (2.11–3.40)^‡^	2.47 (1.96–3.10)^‡^	2.59 (2.05–3.28)^‡^	1.58 (1.26–1.98)^‡^	2.11 (1.68–2.65)^‡^	2.31 (1.85–2.88)^‡^
Onset–to–door time								
≤ 4.5 h	1.0	1.0	1.0	1.0	1.0	1.0	1.0	1.0
>4.5 h	1.02 (0.93–1.12)	0.94 (0.85–1.03)	0.97 (0.88–1.07)	0.94 (0.80–1.04)	1.02 (0.93–1.12)	0.99 (0.90–1.08)	1.01 (0.92–1.11)	1.06 (0.96–1.16)
Door–to–image time								
≤ 1 h	1.0	1.0	1.0	1.0	1.0	1.0	1.0	1.0
>1 h	1.11 (0.98–1.27)	1.07 (0.93–1.23)	1.08 (0.95–1.23)	1.14 (0.99–1.32)	1.16 (1.02–1.32)*	1.06 (0.92–1.22)	1.15 (1.01–1.31)*	1.17 (1.01–1.34)*
Medical history								
Smoker								
Current smoker	0.99 (0.88–1.12)	0.98 (0.86–1.12)	0.94 (0.83–1.06)	1.06 (0.92–1.21)	0.98 (0.87–1.11)	1.06 (0.93–1.22)	0.99 (0.88–1.11)	1.02 (0.89–1.16)
Ex–smoker	1.08 (0.95–1.22)	1.14 (1.00–1.30)	0.91 (0.81–1.03)	1.05 (0.92–1.20)	0.97 (0.85–1.09)	1.06 (0.93–1.20)	1.01 (0.89–1.14)	1.16 (1.02–1.32)*
Non–smoker	1.0	1.0	1.0	1.0	1.0	1.0	1.0	1.0
Atrial fibrillation/flutter								
Yes	1.29 (1.11–1.50)^‡^	1.18 (1.00–1.39)	1.02 (0.88–1.18)	1.11 (0.95–1.30)	1.05 (0.91–1.22)	1.01 (0.86–1.18)	1.11 (0.90–1.28)	1.20 (1.02–1.42)*
No	1.0	1.0	1.0	1.0	1.0	1.0	1.0	1.0
CCI score								
0	1.0	1.0	1.0	1.0	1.0	1.0	1.0	1.0
1	1.18 (1.02–1.37)*	1.30 (1.11–1.52)^†^	1.19 (1.03–1.38)*	1.29 (1.10–1.51)^†^	1.20 (1.03–1.39)*	1.12 (0.95–1.31)	1.04 (0.90–1.21)	1.13 (0.96–1.32)
2	1.35 (1.16–1.56)^‡^	1.49 (1.28–1.74)^‡^	1.37 (1.18–1.58)^‡^	1.40 (1.20–1.64)^‡^	1.35 (1.17–1.57)^‡^	1.22 (1.05–1.43)*	1.25 (1.08–1.45)^†^	1.11 (0.95–1.30)
≥3	1.78 (1.57–2.03)^‡^	1.73 (1.51–1.98)^‡^	1.68 (1.48–1.91)^‡^	1.58 (1.38–1.81)^‡^	1.51 (1.33–1.72)^‡^	1.26 (1.10–1.45)^†^	1.28 (1.13–1.46)^‡^	1.19 (1.04–1.37)*
Medical facility type								
Tertiary general hospital	1.0	1.0	1.0	1.0	1.0	1.0	1.0	1.0
General hospital	1.14 (1.05–1.24)^†^	1.07 (0.98–1.16)	1.03 (0.95–1.12)	1.09 (1.00–1.19)*	1.06 (0.98–1.15)	1.01 (0.93–1.10)	1.06 (0.98–1.15)	1.05 (0.97–1.14)
Post–stroke antithrombotic medication								
No medication	1.0	1.0	1.0	1.0	1.0	1.0	1.0	1.0
Antiplatelet medication	0.70 (0.53–0.91)^†^	0.65 (0.50–0.85)^†^	0.62 (0.47–0.81)^‡^	0.79 (0.61–1.03)	0.77 (0.59–1.01)	0.50 (0.38–0.64)^‡^	0.79 (0.61–1.03)	0.64 (0.49–0.83)^‡^
Anticoagulant medication	0.64 (0.47–0.87)^†^	0.80 (0.59–1.08)	0.76 (0.56–1.03)	0.86 (0.63–1.17)	0.78 (0.57–1.06)	0.51 (0.38–0.69)^‡^	0.90 (0.66–1.22)	0.52 (0.39–0.71)^‡^
Antiplatelet & Anticoagulant medication	0.77 (0.58–1.02)	0.73 (0.55–0.97)*	0.79 (0.60–1.05)	0.93 (0.70–1.25)	0.95 (0.71–1.25)	0.55 (0.41–0.72)^‡^	0.75 (0.57–0.99)*	0.61 (0.46–0.81)^‡^

*Values are presented as HR (95% CI), HR = Hazard Ratio, 95% CI = 95% confidence intervals*.

*IVT, intravenous thrombolysis; EMS, emergency medical services; NIHSS, National Institutes of Health Stroke Scale; CCI, Charlson comorbidity index*.

*p < 0.05 significance.

*^†^p < 0.01 significance*.

*^‡^p < 0.001 significance*.

Compared with non-IVT, IVT given within 2 h of the symptom onset was associated with decreased 3-month (HR = 0.62; 95% CI: 0.52–0.74, *p* < 0.001), 1-year (HR = 0.82; 95% CI: 0.69–0.98, *p* < 0.05), 2-year (HR = 0.80; 95% CI: 0.67–0.95, *p* < 0.05), and 4-year (HR = 0.63; 95% CI: 0.53–0.75, *p* < 0.001) mortality in pre-2015, and decreased 3-month (HR = 0.53; 95% CI: 0.44–0.64, *p* < 0.001), 1-year (HR = 0.66; 95% CI: 0.55–0.79, *p* < 0.001), 2-year (HR = 0.77; 95% CI: 0.64–0.93, *p* < 0.01), and 4-year mortality (HR = 0.77; 95% CI: 0.64–0.93, *p* < 0.01) in post-2015.

IVT administered between 2 and 4.5 h after the symptom onset was associated with decreased 3-month (HR = 0.72; 95% CI: 0.60–0.87, *p* < 0.001), 1-year (HR = 0.83, 95% CI: 0.68–1.00, *p* < 0.05) and 2-year mortality (HR = 0.82; 95% CI: 0.68-0.99, *p* < 0.05), but not decreased 4-year mortality (HR = 0.93; 95% CI: 0.75–1.12, *p* >0.05) when compared with non-IVT in pre-2015, and decreased 3-month (HR = 0.70; 95% CI: 0.58–0.86, *p* < 0.001), 1-year (HR = 0.80; 95% CI: 0.66-0.98, *p* < 0.05), 2-year (HR = 0.77; 95% CI: 0.63-0.94, *p* < 0.01), and 4-year mortality (HR = 0.63; 95% CI: 0.51–0.77, *p* < 0.001) in post-2015.

Additionally, MT significantly decreased 1-year mortality by 12% (HR = 0.88; 95% CI: 0.70–1.10, *p* < 0.05) in pre-2015 when compared with non-MT. However, MT did not significantly decrease 3-month, 2-year, or 4-year mortality (*p* > 0.05) in pre-2015. Nevertheless, MT decreased 3-month (HR = 0.69; 95% CI: 0.56–0.84, *p* < 0.001), 1-year (HR = 0.75; 95% CI: 0.61–0.92, *p* < 0.01), and 2-year mortality (HR = 0.80; 95% CI: 0.65–0.99, *p* < 0.05) post-2015.

Moreover, compared with no medication, antiplatelets did not show a difference in 2-year (HR = 0.77; 95% CI: 0.59–1.01, *p* >0.05) or 4-year (HR = 0.79; 95% CI: 0.61–1.03, *p* >0.05) mortality pre-2015. However, antiplatelets significantly decreased 2-year (HR = 0.50; 95% CI: 0.38–0.64, *p* < 0.001) and 4-year mortality (HR = 0.64; 95% CI: 0.49–0.83, *p* < 0.001) in post-2015. Furthermore, anticoagulant did not show a difference in 2-year (HR = 0.78; 95% CI: 0.57–1.06, *p* >0.05), and 4-year (HR = 0.90; 95% CI: 0.66–1.22, *p* > 0.05) in pre-2015. By contrast, anticoagulant significantly decreased 2-year (HR = 0.51; 95% CI: 0.38–0.69, *p* < 0.001), 4-year mortality (HR = 0.52; 95% CI: 0.39–0.71, *p* < 0.001) in post-2015.

### Change in stroke care hospital

Table 3 shows the changing trends in hospitals treating patients with AIS in Korea. Between pre- and post-2015, the proportion of MT-capable hospitals increased from 33.3% (67) to 49.6% (123), and only IVT-capable hospitals decreased from 37.8% (76) to 23.4% (58). The proportion of tertiary hospitals decreased from 20.9% (42) to 18.1% (45), and general hospitals increased from 69.7% (140) to 85.9% (213). Supplementary Table 3 shows the distribution of medical facilities by type. While the proportion of IVT-capable tertiary hospitals decreased from 40 (19.9%) to 44 (17.7%), IVT-capable general hospitals increased from 77 (38.3%) to 112 (45.2%). The proportion of MT-capable tertiary and general hospitals increased from 27 (13.4%) to 39 (15.7%) and from 26 (12.9%) to 69 (27.8%), respectively. The number of IVT+MT-capable general hospitals increased from 17 (8.5%) to 58 (23.4%).

**Table 3:** Change of distribution of IVT- and MT-capable hospitals in pre-2015 and post-2015.

	**Pre-2015**		**Post-2015**		**Change of distributio*n* (%)**
Total number of hospitals	201	100%	248	100%	0
IVT-capable hospitals	143	71.1%	181	73.0%	+1.9
MT-capable hospitals	67	33.3%	123	49.6%	+16.3
IVT+MT-capable hospitals	67	33.3%	123	49.6%	+16.3
Only IVT–capable hospitals	76	37.8%	58	23.4%	−14.4
General hospitals	140	69.7%	213	85.9%	+16.2
Tertiary hospitals	42	20.9%	45	18.1%	−2.8
Hospitals with stroke unit	66	32.8%	79	31.9%	−0.9

*IVT, intravenous thrombolysis; MT, mechanical thrombectomy*.

### Development of ICH after MT

The proportion of patients developing ICH after MT is illustrated in Supplementary Table 4. In 2013, 14 (9.8%) patients got the development of ICH, 10 (8.8%) in 2014, 39 (7.8%) in 2016, and 38 (7.1%) in 2018.

## Discussion

We investigated the change of short- and long-term mortality in patients with acute AIS according to the changes in stroke management guidelines and health insurance coverage since 2015. Thrombolysis has reduced the long-term mortality of patients with AIS and improved their quality of life since 2015. On Cox analysis, active early management such as thrombolysis and thrombectomy in patients with AIS reduced the mortality in post-2015 compared to pre-2015. Thrombolysis reduced the 4-year mortality of patients with AIS as well as 3-month, 1-year, and 2-year since 2015 (Table 2). Although the National Institute of Neurological Disorders and Stroke trials obtained better functional outcomes after IVT (5, 6), thrombolysis failed to reduce mortality at the 12-month follow-up. The third International Stroke Trial also failed to reduce mortality after thrombolysis at the 18-month follow-up (6). However, these randomized selected patients were not representative of all routine clinical practices. A similar result to our previous report was shown that thrombolysis and thrombectomy failed to reduce the long-term mortality before 2015 (2). A Danish study on register-based nationwide data showed that thrombolysis significantly reduced long-term mortality by up to 7 years in patients with AIS (7). Because thrombolysis decreased the incidence of stroke complications such as cardiopulmonary and venous thrombosis and morbidity by lowering the final infarct size, thrombolysis as initial management for patients with AIS reduced the long-term mortality (7, 8).

In Korea, HIRA changed several guidelines for acute AIS management in 2015. Although thrombolysis was only recommended as active AIS management in the acute stage before 2015, HIRA announced the new indication of MT and extended the insurance coverage in 2015. Stroke academy also recommended thrombectomy as effective management in patients with acute AIS at the same time. In Korea, these changes of thrombectomy guidelines as active management in the acute stage successfully reduced the short- and long-term mortality for patients with AIS. Thrombectomy has reduced the 3-month, 1-year, and 2-year mortality in patients with AIS since 2015 (Table 2).

Since 2015, the expansion of accessibility to bridging therapy and the increasing number of comprehensive stroke centers supporting high-quality care and thrombectomy have led to lower mortality (17, 18). In a report on the United States, the proportion of patients with AIS receiving IVT also decreased from 46–24% (*p* < 0.001), and those receiving MT also increased from 1.1–2.3% (19). These changes reduced in-hospital mortality from 15–13% in the United States. Since the expansion of the insurance criteria by HIRA according to the 2015 AHA guideline, MT-capable hospitals and patients with AIS treated with MT increased from 2013 to 2018 in Korea (Table 3). The patients receiving IVT decreased in post-2015, while those treated with MT significantly increased (Table 1). Additionally, the number of MT-capable and IVT+MT-capable general hospitals easily accessible to patients with AIS in the acute stage significantly increased from pre- to post-2015 (Supplementary Table 3). Inadequate indication of reperfusion therapy makes the prognosis or the survival rate worse. In a previous study, hemorrhagic transformation occurs in about 29% of patients (87 out of 299), and it is reported that 4% of them develop ICH (20). The risk of hemorrhagic infarction after MCA occlusion and subsequent MT is primarily determined by factors affecting infarct severity (20). In this study, the proportion of ICH development after MT decreased from 9.8% in 2013 to 7.1% in 2018. The incidence of ICH after MT has reduced due to well-established adequate selection of reperfusion therapy and development of post-stoke management since 2015 (Supplementary Table 4). Since 2015, according to the guidelines of HIRA and Korea stroke academy, if more than 1/3 of the non-contrast CT images had already progressed, severe cerebral edema or hemorrhage was suspected; all cases were excluded as reimbursement of insurance. These changes in guidelines lowered the likelihood of hemorrhagic transformation or ICH in Korea and improved the life quality and the survival rate during long-term follow-up.

Antithrombotic medications, including antiplatelet or anticoagulant medications, are recommended for nearly all patients with AIS without contraindications (21). Large-artery atherosclerosis is Korea's most common stroke subtype, and cardioembolic is the third most common subtype (22). Therefore, the proportion of medication use in these stroke subtypes varies according to race, nation, and region. In our study, the percentage of patients with AIS using antithrombotic medications increased significantly from pre-2015 to post-2015 (85.9 to 95.4%) (Table 1). The reason many patients who received both anticoagulant and antithrombotic drugs seems to be the concurrent use of aspirin, which has increased because of the high rate of large-artery atherosclerosis in Koreans and the accompanying lesion of intracranial stenosis according to TOAST classification (22). The proportion of the patients with AIS with atrial fibrillation in pre- and post-2015 was similar at 16 and 16.2% in this study, and the proportion of the patients who received anticoagulants was similar, about 16.8~17.3%. Proper antithrombotic medications affected prognosis of patients with AIS. Especially, the proper use of anticoagulants in patients with AIS with atrial fibrillation and the increase in the proper post-stroke antithrombotic medication reduced long-term mortality in Korea. Since 2015, the guideline and insurance coverage of new oral anticoagulant (NOAC) have been changed for patients with atrial fibrillation in Korea. HIRA announced the extension of insurance coverage of NOAC for patients with AIS with atrial fibrillation, and the stroke academy also changed the guideline of anticoagulants of cardioembolic stroke in Korea (23). HIRA announced the full reimbursement of NOAC in 2015, and overall oral anticoagulation prescription in total atrial fibrillation in Korea has increased annually (3.72%) since 2015, compared to before 2015 (1.16%) (23). Most warfarin medications have been replaced with NOAC since 2015, and these changed anticoagulant guidelines decreased the 2- and 4-year mortality on Cox analysis. A meta-analysis showed that DOACs were superior to warfarin in preventing a combination of stroke and systemic embolism with additional risk factors in stroke in patients with atrial fibrillation (24). In our data, the proportion of post-stroke DOAC use for patients with AIS increased significantly from pre- to post-2015 (0.8 to 13.8%). Considering the proportion of atrial fibrillation in patients with AIS was about 16% during the same period in Korea, increasing the usage of NOAC seems to improve the quality of stroke cases and lower the long-term mortality in Korea.

This study had some limitations. No information about the etiology of the strokes, such as the Trial of Org 10172 in Acute Stroke Treatment criteria, computed tomography or magnetic resonance imaging, or information about the occlusion site was available because of no data of these sources in Korea stroke registry (22); therefore, the stroke severity could not be evaluated using brain imaging. Instead, we used NIHSS and CCI: including the history of diabetes mellitus, myocardial infarction, moderate or severe renal disease, and hypertension (25). Additionally, no data about the cause of death were available, and studies on the long-term outcomes after AIS generally report mortality without the cause of death. However, CCI and NIHSS were used as the severity of patients with AIS, and Cox analysis used the adjusted model of risk factors. In addition, all scales measured at discharge were used, since the functional outcomes of all the patients were not measured with the same functional scale. We adjusted the good or poor outcome according to the definition of previous studies. Furthermore, since this study used data collected during a fixed period provided by the country, it was impossible to unify the month of data collection. Data drawn from different months may be a confounding factor; therefore, a follow-up study that unifies them is needed. However, this registry was surveyed in restricted and well-controlled environments in pre-selected hospitals so that this bias would not affect the large impact. A substantial proportion of cases were excluded due to missing data, which may compromise this manuscript's representativeness; the study had to be conducted based on clear onset patients, and all the patients with an unknown onset were excluded. The patients missing the NIHSS, a representative clinical severity scale, were excluded (35.8%). mRS at discharge on post-2015 was poor than that of pre-2015. Despite good functional outcomes leads lower mortality, there was still under the debate on study of long-term mortality of stroke. The patients with AIS with poor functional outcomes would have died in short-term follow-up, even if they survived active treatment in the early stages (9, 26, 27). We did not proceed with direct comparison between each treatment group of pre-2015 and that of post-2015, because it is insufficient to explain our theory and opinion. The guideline, indication, procedure method, and insurance coverage of thrombectomy, medication, acute managements since 2015 have dramatically changed. On logistic regression analysis, there was a statistical difference of mortality on post-2015 compared with that of pre-2015 (Supplementary Table 5). We thought whether the outcome of death according to each acute management, such as thrombolysis, thrombectomy, and anti-thrombotic medication, had statistical significance in each time period or not was more important than the difference of the death according to treatment between pre- and post- 2015. So, the cox analysis that we conducted is more appropriate for evaluation of the short- and long-term mortality according to treatment. This comparison is considered to be more accurate for looking for change in efficacy of same treatment between different eras.

In conclusion, changes in AIS management and the health insurance care system since 2015 have significantly impacted short- and long-term mortality of patients with AIS. Since 2015, IVT has significantly reduced short- and long-term mortality (3-month, 1-year, 2-year, and 4-year), and MT has significantly reduced the 3-month, 1-year, and 2-year mortality. With the increasing use of NOAC from 2013 to 2018, post-stroke antithrombotic medication has significantly lowered the 2-year and 4-year mortality since 2015.

## Data availability statement

The data analyzed in this study was obtained from the Health Insurance Review and Assessment Service (HIRA; https://opendata.hira.or.kr/home.do), the following licenses/restrictions apply: Requests to access the datasets must be approved by HIRA. Requests to access these datasets should be directed to HIRA, globalhira@hira.or.kr.

## Ethics statement

The studies involving human participants were reviewed and approved by Research Ethics Committee of Soonchunhyang University Hospital. Written informed consent for participation was not required for this study in accordance with the national legislation and the institutional requirements.

## Author contributions

JSO: study concept and design. S-WP, JYL, MRL, and JSO: analysis and interpretation of data and researched literature. All authors co-wrote and revised the article for intellectual content as well as provided approval for article submission.

## Funding

This research was supported by Soonchunhyang University Fund. This research was supported by the Bio and Medical Technology Development Program of the National Research Foundation funded by the Korean government (2020R1F1A1066362) and by the Korean Medical Device Development Fund grant funded by the Korean government (Ministry of Science and ICT, the Ministry of Trade, Industry and Energy, the Ministry of Health and Welfare, Republic of Korea, the Ministry of Food and Drug Safety) (202015X17). The funding sources had no role in the design and conduct of the study; collection, management, analysis, and interpretation of the data; preparation, review, or approval of the manuscript; and decision to submit the manuscript for publication.

## Conflict of interest

The authors declare that the research was conducted in the absence of any commercial or financial relationships that could be construed as a potential conflict of interest.

## Publisher's note

All claims expressed in this article are solely those of the authors and do not necessarily represent those of their affiliated organizations, or those of the publisher, the editors and the reviewers. Any product that may be evaluated in this article, or claim that may be made by its manufacturer, is not guaranteed or endorsed by the publisher.

## Abbreviations

AIS: Acute ischemic stroke
CCI: Charlson Comorbidity Index
DOAC: Direct-acting oral anticoagulant
HIRA: Health Insurance Review &
Assessment Service
ICH: Intracranial hemorrhage
IVT: Intravenous thrombolysis
MT: Mechanical thrombectomy
NIHSS: National Institute of Health Stroke Scale.
