# Supplementary figures and images for: Change of mortality of patients with acute ischemic stroke before and after 2015
Source: Front Neurol. 2022 Aug 24;13:947992. doi: 10.3389/fneur.2022.947992 (PMC9450953; doi:10.3389/fneur.2022.947992)

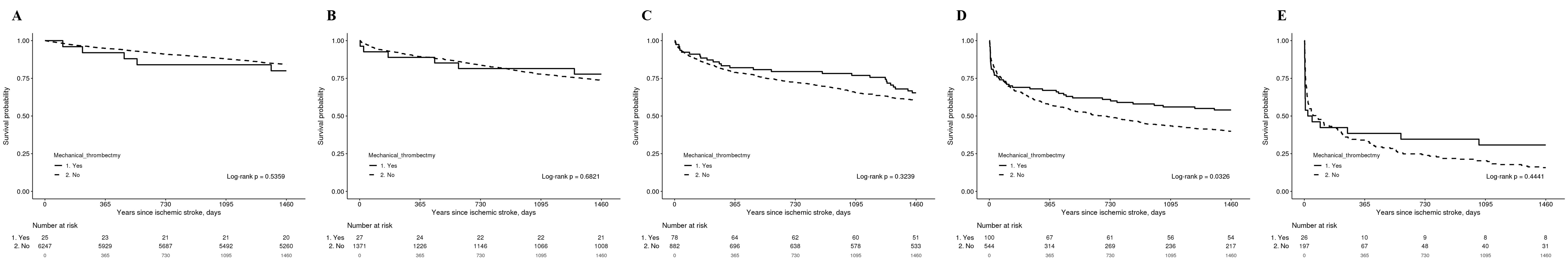

Supplement: Supplementary file 1 [file Image_1.TIF]

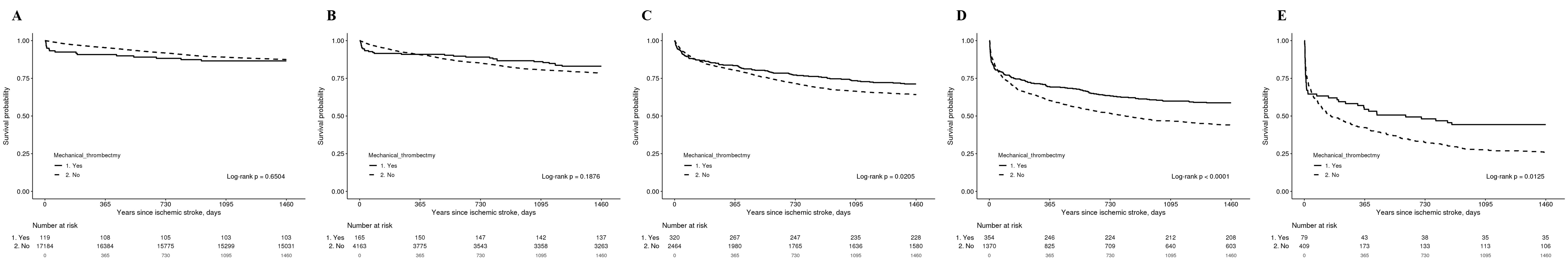

Supplement: Supplementary file 2 [file Image_2.TIF]
