# Supplementary material for: Change of mortality of patients with acute ischemic stroke before and after 2015
Source: Front Neurol. 2022 Aug 24;13:947992. doi: 10.3389/fneur.2022.947992 (PMC9450953; doi:10.3389/fneur.2022.947992)
Supplement: Supplementary file 3 [file Table_1.docx]

***Supplementary Material***

Supplementary Table 1. ICD-10 codes used in the analysis of the study

| Comorbidity | ICD-10 | Weight |
| --- | --- | --- |
| Myocardial infarction | I21.x, I22.x, I25.2 | 1 |
| Congestive heart failure | I09.9, I11.0, I13.0, I13.2, I25.5 ,I42.0, I42.5-9, I43.x, I50.x, P29.0 | 2 |
| Peripheral vascular disease | I70.x, I71.x, I73.1, I73.8-9, I77.1, I79.0, I79.2, K55.1, K55.8-9, Z95.8-9 | 3 |
| Dementia | F00.x-F03.x, F05.1, G30.x, G31.1 |  |
| Chronic pulmonary disease | I27.8-9, J40.x-J47.x, J60.x-J67.x, J68.4, J70.1, J70.3 |  |
| Rheumatologic disease | M05.x, M06.x, M31.5, M32.x-M34.x, M35.1, M35.3, M36.0 |  |
| Pepticulcer disease | K25.x-K28.x |  |
| Mildliver disease | B18.x, K70.0-K70.3, K70.9, K71.3-K71.5, K71.7, K73.x, K74.x, K76.0, K76.2-4, K76.8-9, Z94.4 |  |
| Diabetes without chronic complication | E10.0-1, E10.6, E10.8-9, E11.0-1, E11.6, E11.8-9, E12.0-1, E12.6, E12.8-9, E13.0-1, E13.6, E13.8-9, E14.0-1, E14.6, E14.8-9 |  |
| Diabetes with chronic complication | E10.2-5, E10.7, E11.2-5, E11.7, E12.2-5, E12.7, E13.2-5, E13.7, E14.2-5, E14.7 |  |
| Hemiplegia or paraplegia | G04.1, G11.4, G80.1-2, G81.x, G82.x, G83.0-4, G83.9 |  |
| Moderate or severe renal disease | I12.0, I13.1, N03.2-7, N05.2-7, N18.x, N19.x, N25.0, Z49.0-2, Z94.0, Z99.2 |  |
| Any malignancy | C00.x-C26.x, C30.x-C34.x, C37.x-C41.x, C43.x, C45.x-C58.x, C60.x-C76.x, C81.x-C85.x, C88.x, C90.x-C97.x |  |
| Moderate or severe liver disease | I85.0, I85.9, I86.4, I98.2, K70.4, K71.1, K72.1, K72.9, K76.5-7 |  |
| Metastatic solid tumor | C77.x-C80.x |  |
