# Supplementary material for: Change of mortality of patients with acute ischemic stroke before and after 2015
Source: Front Neurol. 2022 Aug 24;13:947992. doi: 10.3389/fneur.2022.947992 (PMC9450953; doi:10.3389/fneur.2022.947992)
Supplement: Supplementary file 4 [file Table_2.DOCX]

***Supplementary Material***

Supplementary Table 2. Changes in drug use in patients before and after 2015.

| type |  | Pre-2015 (n=9,497) | Post-2015 (n=26,627) |
| --- | --- | --- | --- |
| antiplatelet | Cilostazole | 1,159 (12.2%) | 3,277 (12.3%) |
|  | Clopidogrel | 5,066 (53.3%) | 18,301 (68.7%) |
|  | Aspirin | 6,716 (70.7%) | 21,700 (81.5%) |
|  | Ticlopidine | 388 (4.1%) | 783 (2.9%) |
| anticoagulant | DOAC | 78 (0.8%) | 3,676 (13.8%) |
|  | Warfarin | 1,568 (16.5%) | 1,143 (4.3%) |
