# Supplementary material for: Change of mortality of patients with acute ischemic stroke before and after 2015
Source: Front Neurol. 2022 Aug 24;13:947992. doi: 10.3389/fneur.2022.947992 (PMC9450953; doi:10.3389/fneur.2022.947992)
Supplement: Supplementary file 5 [file Table_3.docx]

***Supplementary Material***

Supplementary Table 3. The distribution by type of medical facility and the number of surgeons and specialists

|  | 2013.06–2013.08 (201 hospitals) | | | 2014.06–2014.08 (189 hospitals) | | | 2016.06–2016.12 (246 hospitals) | | | 2018.06–2018.12 (248 hospitals) | | |
| --- | --- | --- | --- | --- | --- | --- | --- | --- | --- | --- | --- | --- |
|  | IVT | MT | IVT+MT | IVT | MT | IVT+MT | IVT | MT | IVT+MT | IVT | MT | IVT+MT |
| Tertiary hospitals | 40 (19.9%) | 27 (13.4%) | 22 (10.9%) | 41 (21.7%) | 31 (16.4%) | 27 (14.3%) | 42 (17.1%) | 41 (16.7%) | 39 (15.9%) | 44 (17.7%) | 39 (15.7%) | 36 (14.5%) |
| General hospitals | 77 (38.3%) | 26 (12.9%) | 17 (8.5%) | 84 (44.4%) | 16 (8.5%) | 10 (5.3%) | 116 (47.2%) | 57 (23.2%) | 41 (16.7%) | 112 (45.2%) | 69 (27.8%) | 58 (23.4%) |
| Hospitals with Stroke unit | 51 (25.4%) | 34 (16.9%) | 24 (11.9%) | 61 (32.3%) | 32 (16.9%) | 26 (13.8%) | 67 (27.2%) | 61 (24.8%) | 52 (21.1%) | 70 (28.2%) | 62 (25.0%) | 58 (23.4%) |
| Hospitals with Stroke unit certification | 33 (16.4%) | 24 (11.9%) | 16 (8.0%) | 41 (21.7%) | 27 (14.3%) | 22 (11.6%) | 46 (18.7%) | 42 (17.1%) | 36 (14.6%) | 50 (20.2%) | 45 (18.1%) | 42 (16.9%) |
| Neurologist (mean±SD) | 6.3±4.5 | 7.9±6.1 | 8.0±5.7 | 6.1±4.5 | 7.6±4.9 | 7.9±5.2 | 6.7±5.1 | 6.9±5.3 | 7.0±5.3 | 6.9±5.1 | 6.6±4.7 | 6.6±4.8 |
| Neurosurgeon (mean±SD) | 6.7±4.0 | 8.0±5.0 | 8.0±4.9 | 6.7±4.2 | 8.3±4.3 | 8.5±4.6 | 7.6±5.5 | 8.3±6.1 | 8.3±6.1 | 7.2±4.9 | 7.0±4.4 | 7.1±4.5 |
| Rehabilitation Specialist (mean±SD) | 3.2±1.8 | 3.5±2.0 | 3.7±2.0 | 3.2±2.1 | 4.1±2.2 | 4.2±2.2 | 3.4±2.3 | 3.7±2.5 | 3.7±2.4 | 3.3±2.3 | 3.2±2.1 | 3.3±2.2 |

IVT=intravenous thrombolysis; MT=mechanical thrombectomy
