# Supplementary material for: Change of mortality of patients with acute ischemic stroke before and after 2015
Source: Front Neurol. 2022 Aug 24;13:947992. doi: 10.3389/fneur.2022.947992 (PMC9450953; doi:10.3389/fneur.2022.947992)
Supplement: Supplementary file 6 [file Table_4.DOCX]

***Supplementary Material***

Supplementary Table 4. Analysis according to year of patients who got intracerebral hemorrhage of MT after 30 days.

| Year (Number of MT patients) | ICH after MT (30 days) |
| --- | --- |
| 2013 (n=143) | 14 (9.8%) |
| 2014 (n=113) | 10 (8.8%) |
| 2016 (n=503) | 39 (7.8%) |
| 2018 (n=534) | 38 (7.1%) |
