# Supplementary material for: Change of mortality of patients with acute ischemic stroke before and after 2015
Source: Front Neurol. 2022 Aug 24;13:947992. doi: 10.3389/fneur.2022.947992 (PMC9450953; doi:10.3389/fneur.2022.947992)
Supplement: Supplementary file 7 [file Table_5.DOCX]

***Supplementary Material***

Supplementary Table 5. Logistic regression model for the risk of death in 3-month, 1-year, 2-year and 4-year.

|  | 3-month | | 1-year | | 2-year | | 4-year | |
| --- | --- | --- | --- | --- | --- | --- | --- | --- |
|  | OR (95% CI) | P value | OR (95% CI) | P value | OR (95% CI) | P value | OR (95% CI) | P value |
| Pre-2015 | 1.0 |  | 1.0 |  | 1.0 |  | 1.0 |  |
| Post-2015 | 0.85(0.70-1.03) | 0.0904 | 0.80(0.71-0.91) | 0.0005 | 0.84(0.76-0.93) | 0.0006 | 0.87(0.80-0.95) | 0.0012 |
| Onset to treatment time |  |  |  |  |  |  |  |  |
| IVT≤120 min | 0.42(0.29-0.59) | <.0001 | 0.36(0.28-0.46) | <.0001 | 0.35(0.28-0.44) | <.0001 | 0.40(0.33-0.48) | <.0001 |
| 120 min<IVT≤270 min | 0.48(0.32-0.73) | 0.0006 | 0.50(0.38-0.66) | <.0001 | 0.54(0.43-0.68) | <.0001 | 0.58(0.48-0.71) | <.0001 |
| Non-IVT | 1.0 |  | 1.0 |  | 1.0 |  | 1.0 |  |
| Mechanical Thrombectomy |  |  |  |  |  |  |  |  |
| Yes | 0.90(0.63-1.30) | 0.5851 | 0.70(0.53-0.93) | 0.0151 | 0.75(0.58-0.96) | 0.0221 | 0.68(0.55-0.86) | 0.0011 |
| No | 1.0 |  | 1.0 |  | 1.0 |  | 1.0 |  |
| Age (years) |  |  |  |  |  |  |  |  |
| 18–45 | 1.0 |  | 1.0 |  | 1.0 |  | 1.0 |  |
| 46–59 | 2.28(0.53-981) | 0.267 | 2.44(0.97-6.14) | 0.0576 | 2.56(1.24-5.32) | 0.0115 | 2.61(1.53-4.48) | 0.0005 |
| 60–69 | 4.96(1.20-20.45) | 0.0269 | 5.18(2.11-12.73) | 0.0003 | 5.67(2.78-11.57) | <.0001 | 5.30(3.13-8.98) | <.0001 |
| ≥70 | 11.07(2.73-44.96) | 0.0008 | 14.52(5.97-35.33) | <.0001 | 18.07(8.92-36.59) | <.0001 | 21.82(12.97-36.72) | <.0001 |
| Male, sex | 1.0 |  | 1.0 |  | 1.0 |  | 1.0 |  |
| Female, sex | 1.14(0.91-1.43) | 0.2532 | 0.83(0.72-0.96) | 0.0110 | 0.78(0.69-0.88) | <.0001 | 0.74(0.67-0.82) | <.0001 |
| Health insurance type |  |  |  |  |  |  |  |  |
| Health insurance | 1.0 |  | 1.0 |  | 1.0 |  | 1.0 |  |
| Medical aid | 0.62(0.42-0.90) | 0.0131 | 1.00(0.81-1.25) | 0.9751 | 1.19(1.00-1.41 | 0.0540 | 1.43(1.24-1.66) | <.0001 |
| Arrival mode |  |  |  |  |  |  |  |  |
| EMS | 1.0 |  | 1.0 |  | 1.0 |  | 1.0 |  |
| No EMS | 0.57(0.45-0.72) | <.0001 | 0.66(0.58-0.76) | <.0001 | 0.67(0.60-075) | <.0001 | 0.73(0.66-0.80) | <.0001 |
| NIHSS |  |  |  |  |  |  |  |  |
| 1–4 | 1.0 |  | 1.0 |  | 1.0 |  | 1.0 |  |
| 5–7 | 2.31(1.70-3.14) | <.0001 | 1.86(1.56-2.22) | <.0001 | 1.73(1.51-1.99) | <.0001 | 1.73(1.54-1.94) | <.0001 |
| 8–13 | 5.15(3.87-6.85) | <.0001 | 3.65(3.05-4.35) | <.0001 | 3.08(2.66-3.58) | <.0001 | 2.94(2.57-3.36) | <.0001 |
| 14–21 | 9.84(7.28-13.29) | <.0001 | 6.24(5.09-7.65) | <.0001 | 5.37(4.48-6.43) | <.0001 | 4.67(3.93-5.54) | <.0001 |
| 22–42 | 19.08(13.02-27.96) | <.0001 | 14.00(10.30-19.02) | <.0001 | 1.41(10.40-19.33) | <.0001 | 13.45(9.59-18.86) | <.0001 |
| Onset-to-door time |  |  |  |  |  |  |  |  |
| ≤4.5 h | 1.0 |  | 1.0 |  | 1.0 |  | 1.0 |  |
| >4.5 h | 1.14(0.91-1.43) | 0.2496 | 1.11(0.97-1.28) | 0.1368 | 1.05(0.93-1.18) | 0.4333 | 0.98(0.89-1.08) | 0.6610 |
| Door-to-image time |  |  |  |  |  |  |  |  |
| ≤1 h | 1.0 |  | 1.0 |  | 1.0 |  | 1.0 |  |
| >1 h | 1.61(1.17-2.21) | 0.0034 | 1.30(1.0-1.60) | 0.0110 | 1.23(1.04-1.46) | 0.0137 | 1.12(0.97-1.28) | 0.1222 |
| Medical history |  |  |  |  |  |  |  |  |
| Smoker |  |  |  |  |  |  |  |  |
| Current smoker | 0.97(0.70-1.34) | 0.8466 | 0.82(0.67-1.00) | 0.0481 | 0.88(0.75-1.03) | 0.1038 | 0.90(0.79-1.02) | 0.0949 |
| Ex-smoker | 1.37(1.02-1.84) | 0.0374 | 1.28(1.06-1.54) | 0.0094 | 1.25(1.07-1.45) | 0.0046 | 1.12(0.98-1.27) | 0.1009 |
| Non-smoker | 1.0 |  | 1.0 |  | 1.0 |  | 1.0 |  |
| Atrial fibrillation/flutter |  |  |  |  |  |  |  |  |
| Yes | 1.12(0.82-1.53) | 0.4801 | 1.55(1.24-1.93) | <.0001 | 1.56(1.29-1.89) | <.0001 | 1.37(1.16-1.62) | 0.0003 |
| No | 1.0 |  | 1.0 |  | 1.0 |  | 1.0 |  |
| CCI score |  |  |  |  |  |  |  |  |
| 0 | 1.0 |  | 1.0 |  | 1.0 |  | 1.0 |  |
| 1 | 1.87(1.23-2.84) | 0.0035 | 1.31(1.02-1.68) | 0.0357 | 1.25(1.03-1.52) | 0.0244 | 1.27(1.09-1.47) | 0.0020 |
| 2 | 2.42(1.62-3.63) | <.0001 | 1.91(1.51-2.43) | <.0001 | 1.76(1.46-2.13) | <.0001 | 1.64(1.42-1.91) | <.0001 |
| ≥3 | 2.18(1.51-3.15) | <.0001 | 2.27(1.84-2.80) | <.0001 | 2.28(1.93-2.68) | <.0001 | 2.18(1.92-2.48) | <.0001 |
| Medical facility type |  |  |  |  |  |  |  |  |
| Tertiary general hospital | 1.0 |  | 1.0 |  | 1.0 |  | 1.0 |  |
| General hospital | 0.98(0.81-1.19) | 0.8162 | 1.06(0.94-1.20) | 0.3521 | 1.16(1.05-1.29) | 0.0042 | 1.12(1.02-1.22) | 0.0119 |
| Post-stroke antithrombotic medication |  |  |  |  |  |  |  |  |
| No medication | 1.0 |  | 1.0 |  | 1.0 |  | 1.0 |  |
| Antiplatelet medication | 0.21(0.14-031) | <.0001 | 0.30(0.22-0.43) | <.0001 | 0.39(0.28-0.54) | <.0001 | 0.44(0.32-0.60) | <.0001 |
| Anticoagulant medication | 0.29(0.18-0.48) | <.0001 | 0.28(0.19-0.42) | <.0001 | 0.38(0.26-0.56) | <.0001 | 0.47(0.33-0.68) | <.0001 |
| Antiplatelet & Anticoagulant medication | 0.20(0.13-0.32) | <.0001 | 0.28(0.19-0.40) | <.0001 | 0.40(0.28-0.56) | <.0001 | 0.52(0.37-0.72) | <.0001 |
